# A Blockchain-Based Notarization Service for Biomedical Knowledge Retrieval

**DOI:** 10.1016/j.csbj.2018.08.002

**Published:** 2018-08-17

**Authors:** Athina-Styliani Kleinaki, Petros Mytis-Gkometh, George Drosatos, Pavlos S. Efraimidis, Eleni Kaldoudi

**Affiliations:** a Dept. of Electrical and Computer Engineering, Democritus University of Thrace, Kimmeria, Xanthi 67100, Greece; b School of Medicine, Democritus University of Thrace, Dragana, Alexandroupoli 68100, Greece

**Keywords:** Biomedical repositories, Cryptographic techniques, Blockchain, Integrity, Non-repudiation, Versioning

## Abstract

Biomedical research and clinical decision depend increasingly on scientific evidence realized by a number of authoritative databases, mostly public and continually enriched via peer scientific contributions. Given the dynamic nature of biomedical evidence data and their usage in the sensitive domain of biomedical science, it is important to ensure retrieved data integrity and non-repudiation. In this work, we present a blockchain-based notarization service that uses smart digital contracts to seal a biomedical database query and the respective results. The goal is to ensure that retrieved data cannot be modified after retrieval and that the database cannot validly deny that the particular data has been provided as a result of a specific query. Biomedical evidence data versioning is also supported. The feasibility of the proposed notarization approach is demonstrated using a real blockchain infrastructure and is tested on two different biomedical evidence databases: a publicly available medical risk factor reference repository and on the PubMed database of biomedical literature references and abstracts.

## 1 Introduction

Biomedical research and clinical practice rely increasingly on authoritative data gathered and curated in reference biomedical databases. Examples include: clinical databases (registries or academic clinical databases) that hold clinical data on patient cohorts [1]; biomedical databases [2] with current data on pharmaceuticals [3], metabolomics [4], inheritance data and other omics (for example, the rich collection available from the European Bioinformatics Institute at http://www.ebi.ac.uk/services); publication repositories and other medical evidence repositories [5], either general purpose (the most prominent example being PubMed service by the National Library of Medicine, USA) or high evidence quality, such as Cochrane Library reports.

Biomedical references databases are continually updated to include new data sets (e.g. PubMed included ~1.2 M new records in 2017), and are often validated and, if necessary, updated to correct existing data. At any given point in time, these data are heavily accessed by humans (clinicians, patients and researchers alike) and software (via appropriate application programming interfaces) to establish current evidence and inform clinical acts and biomedical research. As such, it is important to ensure that data cannot be manipulated retrospectively and that data ‘consumers’ can have a proof of what data were retrieved from the database at a given point in time as a result of a specific query.

A reliable knowledge retrieval service has to fulfill at least the following two important requirements; integrity and non-repudiation. Integrity, means that the query and the retrieved data cannot be modified (either by accident or deliberately), once the retrieval operation completes. Non-repudiation, in this context means that given any past retrieval operation, the knowledge retrieval service cannot validly deny that the exact data have been provided by the service as a response to the given query at the specific time.

To satisfy the above requirements we propose a solution that is based on blockchain infrastructure concepts and tools. In particular, we proposed the use of blockchain technology to create smart digital contracts to seal the query and the respective results each time a third-party requests knowledge from a biomedical database. This paper builds on our earlier presentation of a first proof of concept [6] and extends the propose service to support evidence data versioning. In addition, the cost for several variations of the blockchain operations are examined. The proposed approach is demonstrated on the Ethereum blockchain platform [7] with a notarized retrieval service for two representative medical knowledge databases, the CARRE risk factor reference repository [8] and the PubMed MEDLINE database [9]. The notary service can be adapted in a straightforward manner to support any other biomedical data sources.

The notarization problem addressed in this work is defined as follows:

A data consumer submits a query to retrieve a response from a biomedical data repository. A notary service acts as the mediator of the transaction between the data consumer and the repository. The data consumer receives the requested medical information and, in addition, an assurance that the following core and optional requirements are satisfied*:*

• Data integrity. The integrity of the medical knowledge retrieval operation, including the query and the retrieved knowledge must be assured.
• Database non-repudiation. The service cannot deny the transaction.
• Data consumer non-repudiation [Optional]. The client cannot deny the transaction.
• Data versioning [Optional]. The service keeps track of the different responses that have been given to the same query, as the contents of the database gradually evolve.

The main results of this work are:

• Two blockchain-based schemes that solve the above defined notarization problem. The first one, the basic scheme, is a rewrite of the basic scheme of our previous work [6]. The basic scheme supports data consumer non-repudiation in addition to the core requirements. The second one, the versioning scheme, supports versioning of the responses in addition to the core requirements.
• A prototype implementation of the proposed schemes in the Ethereum blockchain infrastructure.
• A cost analysis of the Ethereum-based computational operations. Several variations of schemes that make use of the computational capabilities of smart contracts in order to reduce the overall computational cost of the blockchain-based operations, are examined.
• A *lightweight wrapper* that applies the notarization service on two real biomedical databases, the CARRE risk factor reference repository and the PubMed MEDLINE database, and a unified Web UI that is used as a front-end to demonstrate the service.

The rest of this work is organized as follows: Background and Related work are given in Section 2. The notary service along with the blockchain-based schemes are described in Section 3. In Section 4, a cost analysis of the blockchain-based operations is presented. The implementation of the service prototype is given in Section 5. The paper concludes with a discussion in Section 6.

## 2 Background & Related Work

### 2.1 Integrity and Non-repudiation

Data integrity and non-repudiation are well studied topics. A recent survey paper [10] lists and compares different existing methods in order to achieve integrity, authenticity, non-repudiation and proof of existence. Furthermore, the authors of the systematic review [11] provide a comprehensive and structured overview about security requirements and solutions in the area of cloud computing. Accordingly, some other interesting survey papers, in the field of the distributed large-scale data processing in MapReduce [12] and the vehicular ad hoc networks (VANETs) [13], review the current security and privacy aspects on these technologies.

Commonly used methods to ensure *data integrity* are to backup the data, to employ checksums techniques or to use cryptographic hash functions [14]. The most common method uses cryptographic hash functions that have as input arbitrary length data and as output a fixed sized sequence of bits. These are one-way functions, i.e., it is computationally infeasible to compute the input from the output, and they are deterministic, i.e., a specific input always provides the same output, and a slight change of the input results in a completely different output. Thus, to ensure the integrity of a message, a cryptographic hash function is used to compute the hash value of the message. At a later time, the integrity of the message can be checked by comparing the initial, stored hash value with the hash value that is provided by the same cryptographic hash function on the alleged message.

One of the most common techniques to deal with *non-repudiation* are digital signatures [14], the analogue of a handwritten or manual signature. The sender signs the message or the hash value of the message that is produced by a cryptographically secure hash function. Digital signatures are implemented using asymmetric cryptography, that uses a public-private pair of keys. To ensure non-repudiation, the sender signs the message with the private key and the receiver uses the sender's public key to validate this signature. Assumed that the private key is kept secret, it is computationally infeasible for any third party to alter the signed message without invalidating the signature. A problem that occurs is that when someone uses the public key to validate the signature of a message there is not a way to ensure that the public key belongs to a specific identity. For this purpose, a trusted third-party (usually a certification authority) is required to certify that a specific public key belongs to a specific person. Consequently, digital signatures can be used for protection against non-repudiation.

Although there is a large amount of work on data integrity and non-repudiation, the advent of the blockchain infrastructures and especially the recent emergence of smart contracts technology opens new perspectives. The existing methods for data integrity and non-repudiation can be combined with the features of blockchains like robustness, traceability and cost-effectiveness as well as their decentralized applications (Dapps).

### 2.2 Blockchain Technology

Blockchain is a distributed, incorruptible transaction management technology without one single trusted party. The first blockchain was proposed for and implemented in Bitcoin [15], a distributed infrastructure where users can make financial transactions without the need of a regulator (e.g. a bank). Nowadays, other blockchain infrastructures are emerging, for example the Ethereum [7], where everyone can participate in the blockchain generation, and the Hyperledger Fabric [16], where only approved parties can post to the blockchain.

In a blockchain, each new transaction is broadcasted to a distributed network of nodes; once all nodes agree the transaction is valid, the transaction is added to a block. Every block contains a timestamp and the hash of the previous block and the transaction data, thus creating an immutable, append-only chain. Copies of the entire blockchain are maintained by each participating node.

Some blockchain infrastructures, like the Ethereum^1^, support smart contracts, which are immutable computer codes running on top of a blockchain. The functions within a contract and can be invoked in the context of blockchain transactions.

**Fig. 1:**
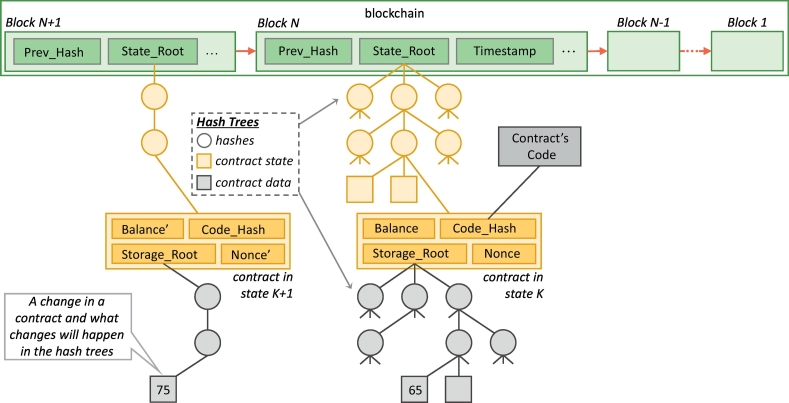
An abstract overview of the implementation of a blockchain and its smart contracts inside the blocks.

An abstract overview of the implementation of a blockchain and its blocks is shown in Fig. 1. Within each block, transaction data are coded into hash trees (Merkle Particia trees [17]) that have a ‘root hash’ that refers to the entire tree; leaf nodes (shown with a square symbol in Fig. 1) correspond to data blocks, while non-leaf nodes (shown with a circle symbol in Fig. 1) correspond to cryptographic hashes of the child nodes. Data on the contract is held within each leaf node; this includes another hash tree that stores contract data (‘Storage Root’), the hash of the contract code (‘Code Hash’), number of transactions sent from the contract (‘Nonce’), and the financial balance (‘Balance’). When there is a change in a contract, the hash tree only stores this change and simply points back to the previous tree for all other contract data.

Blockchains infrastructures charge for each transaction a fee proportional to the computational burden that the execution will impose on the blockchain. This fuel is known as ‘gas’.

A recent systematic review on current state, limitations and open research on blockchain technology [18] discusses a number of blockchain applications that extend from cryptocurrency to Internet of things, smart contracts, smart property, digital content distribution, Botnet, and P2P broadcast protocols.

### 2.3 Blockchain Applications in the Biomedical Domain

Currently, there is considerable optimism that blockchain technology will revolutionize the healthcare industry [19], and there are review articles that thoroughly describe the advantages and challenges of using blockchain technologies in the biomedical domain ([20,21]). Major advantages include [20] the ability to support (a) decentralized data management (e.g. when different healthcare stakeholders need to access patient data); (b) immutable audit trails, implementing the only read and write function for medical data preventing tampering; (c) data provenance, where the origins of the data are traceable, e.g. in the case of patient consent; (d) robustness and availability, highly important to life critically medical data; and (e) security and privacy.

A major application of the blockchain technology in biomedical domain is the field of electronic health records (EHR) which consists of fragments of clinical data related to the patient as generated and maintained by healthcare providers. Such applications include use of blockchain technologies for EHR integration [22], sharing and access control [[23], [24], [25]], preservation [26] and overall management [27,28]. Other application areas on patient data address personal data and services; in particular personal health records generated and maintained by the patient [29], and mobile or other personal ehealth applications [30]. Another interesting field are healthcare services logistics, including medical insurance transactions [31] and drug supply [32]. Furthermore, blockchain technology can also be applied in clinical trial management, with emphasis on participant consent management [33] and privacy preservation [34]. To the best of our knowledge, there is no other work exploiting blockchain technology for managing biomedical evidence data integrity and non-repudiation, other than the preliminary presentation of the proof of concept [6] of the solution described in this paper.

## 3 The Query Notary Service

To support integrity and non-repudiation in biomedical evidence retrieval, we propose a lightweight wrapper for conventional databases that uses blockchain technology to offer database query notary services to data consumers (humans and programs alike). The proposed notary service administers contracts that seal a query to a database and the retrieved results. The service offers irrevocable proof of data retrieved by a specific query placed by a specific consumer, thus establishing query transaction integrity and non-repudiation. Thus, the proposed system assures that the data consumer is protected against a service that may accidentally or intentionally try to repudiate or alter a past query transaction.

**Fig. 2:**
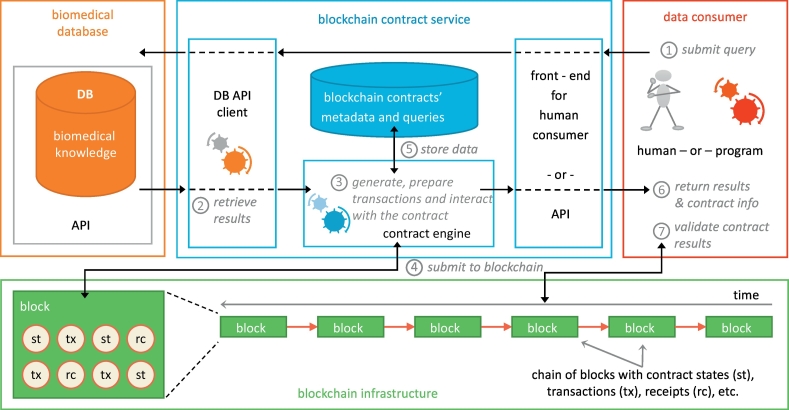
The general architecture of our query notary service.

The overall architecture is presented in Fig. 2. The proposed component is the blockchain contract service that acts as a mediator between conventional biomedical databases and data consumers. The structure of the database may follow any model, from relational to graph databases (e.g. SQL, NoSQL databases, or even RDF repositories).

The proposed notary service exhibits three computational layers: (a) a data consumer front-end, which can be either an interface for human data consumers or an application programming interface (API) for 3rd party programs that request data from a biomedical database; (b) an interface to communicate with biomedical database interfaces, which is specific to each database API; and (c) the contract engine, which collates the query and retrieved results data together with the consumer, generates and prepares transactions, and manages contracts and their metadata.

In this work, we present two distinct functional schemes for the implementation of the notarization service: one that realizes a query-response ledger (*basic scheme*) and one that allows for data versioning (*versioning scheme*). The functionalities of the two schemes are summarized in Table 1. Both schemes satisfy the core requirements of the notary problem. Additionally, the basic scheme supports data consumer non-repudiation, whereas the versioning scheme offers data versioning.

**Table 1:** Functionalities of the two proposed schemes for the database query notary service.

Functionalities	Schemes	
	Basic	Versioning
Data integrity	✓	✓
Database non-repudiation	✓	✓
Data consumer non-repudiation	✓	–
Data versioning	–	✓

### 3.1 The Basic Scheme

**Fig. 3:**
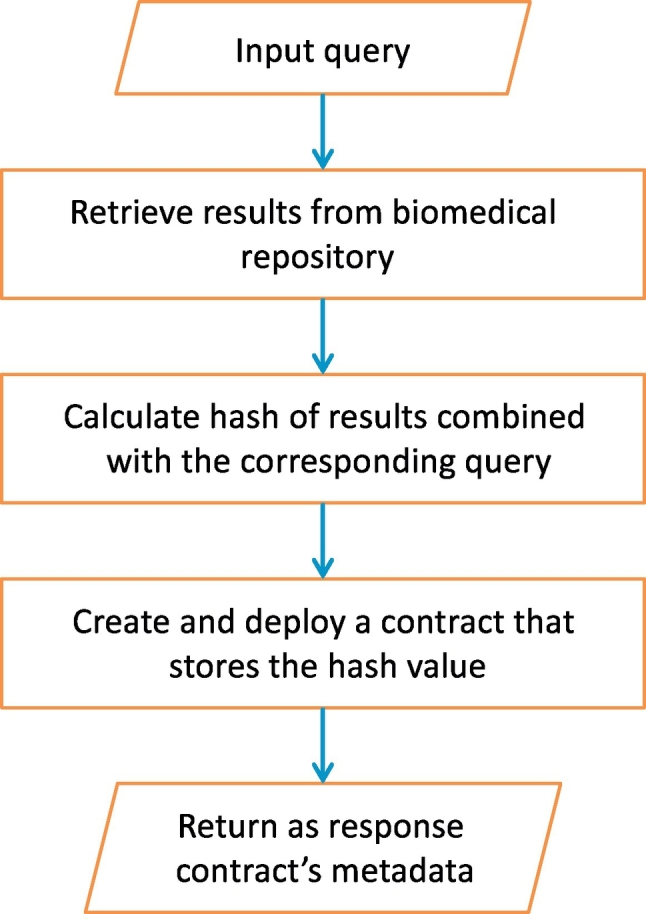
The workflow of the basic scheme when a new contract is deployed per query.

In this scheme, for each query request the notary service generates and deploys a new contract to a blockchain infrastructure. The workflow of this case is shown in Fig. 3 and is described as follows. First, the data consumer front-end undertakes the communication with the party placing the query to the database. In this basic version, the query is forwarded to the database application programming interface (API) via the database API client. As an added-value, the query can also be signed by a public key infrastructure to verify later the identity of the data consumer. This ensures non-repudiation of the data consumer. The API client places the query via the database API and retrieves the results; (signed) query and results are forwarded to the contract engine. Subsequently, these data are hashed (e.g. using the Keccak-256 hash algorithm) and the hash is included in a smart contract that is deployed to a blockchain infrastructure. The contract is written in the Solidity language [7] and its source code is shown in Contract 1.

**Figure:**
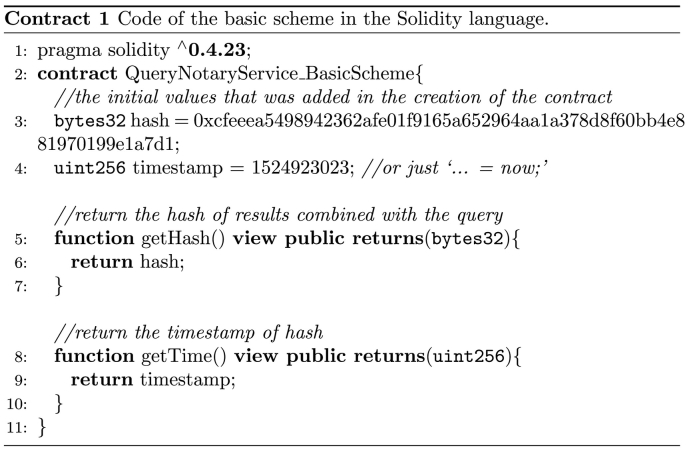

The contract generation engine then returns the query results to the data consumer via the front-end, accompanied by the smart contract's metadata (i.e., contract's address on the blockchain and its application binary interface (ABI)) to interact with the contract, and the (signed) query and its results. A respective entry is also made into the local contract database. The packet returned to the data consumer contains also database certification information to verify the identity of the database and thus ensure query transaction non-repudiation; database identity can, for example, represented by the database blockchain public key signed by a digital certification authority. The consumer archives the query transaction (query and signed response) in a local database for future reference.

At any later time, the data consumer or any third party can verify the query transaction dataset by retrieving the respective contract from the blockchain infrastructure and comparing the retrieved hash of the original data with a new hash of the alleged (signed) query and respective results.

### 3.2 The Versioning Scheme

This scheme satisfies the core requirements of the notary problem and additionally supports versioning of the retrieved data. The key feature of the scheme is that instead of using a smart contract for each submitted query, only a single smart contract is created. This smart contract implements a data structure, which serves as an index of the queries and their corresponding results. Each query is represented with a hash value of the actual query and the hash values of the corresponding results since the day of its creation. Additionally, a timestamp that represents the insertion time of the corresponding contract is assigned to each hash value of the query results pair.

**Figure:**
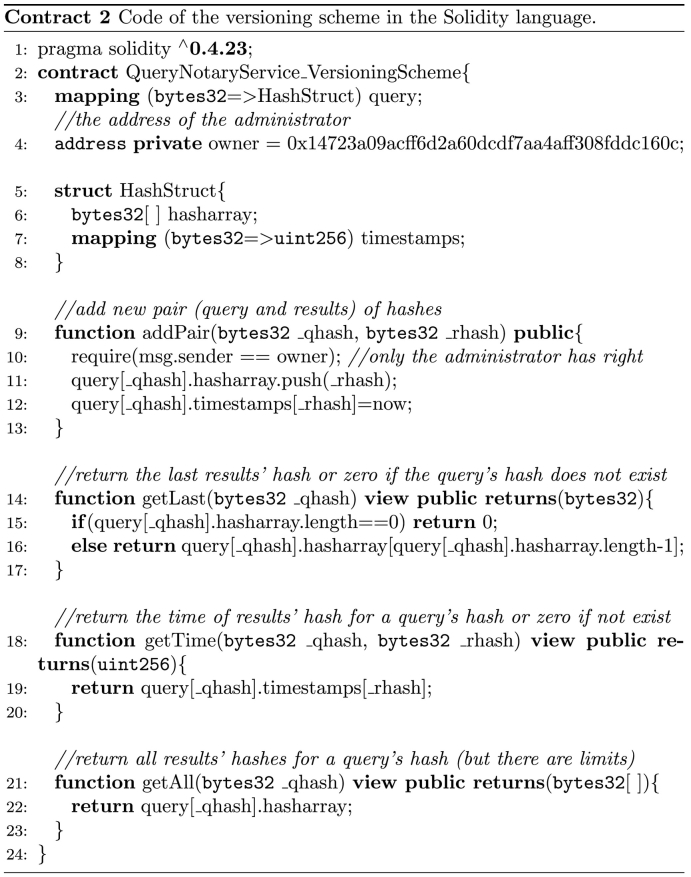

**Fig. 4:**
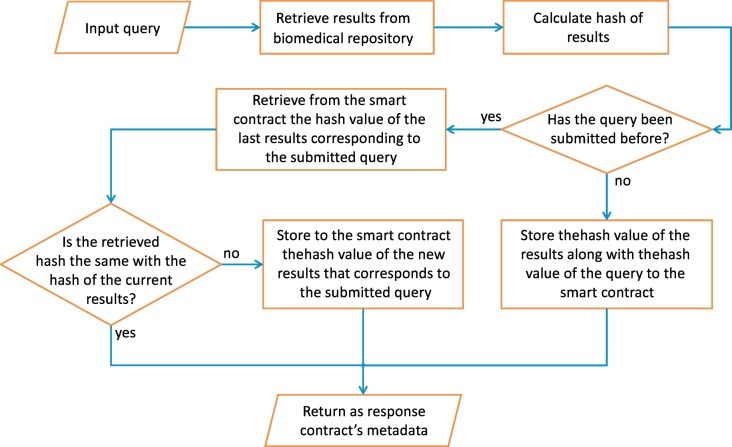
The workflow of the versioning scheme that supports data versioning of query results using a single smart contract.

In the first step of this approach, the contract engine deploys to a blockchain infrastructure only a smart contract that implements the mentioned data structure and the logic of the required functionalities in order to support version control of the query results. The source code of this contract in the Solidity language is shown in Contract 2. After the deployment of the initial smart contract, the notary service is ready to receive queries from the data consumers. The workflow of the versioning scheme is shown in Fig. 4 and considers two different circumstances:

1. *The query has NOT been submitted before.* The hash value of the query and the hash value of the results to the corresponding query are added to the smart contract by the contract engine. Also the query is stored into the local storage database.
2. *The query has been submitted before.* The contract service interacts with the smart contract by calling the appropriate function that will return the hash value of the last results to this query. The contract engine compares the hash value that is retrieved from the smart contract with the hash value of the current results retrieved via the database API. In this case, there are two possible sub-cases:
  (a) *The hash values are NOT the same.* The contract engine interacts with the smart contract and for this query adds the hash value of the new results that are retrieved via the database API. In this way, the smart contract contains all the hash values of the results to a query and it can identify the most recent of them.
  (b) *The hash values are the same.* In this case, the contract engine does not need to add any information to the smart contract.

In all cases, the contract engine returns as a response the query results, accompanied by the query and the smart contract's metadata (i.e., contract's address on the blockchain and its application binary interface (ABI)) that is the same and independent from the query and the query results. A respective entry of hash values for the cases (1) and (2.a), accompanied with the actual queries, is also inserted into the local contract database for performance and logging reasons of the notary service. At any later time, the data consumer or any third party can retrieve the hash values of all the results in a chronological order or just the hash value of the most recent results to a query, accompanied with the representative timestamps of the insertions to the smart contract.

## 4 Cost Analysis Experiments

A series of experiments were conducted in order to analyze the cost of the blockchain-related operations of various realizations of the proposed schemes. The experiments were performed on the Remix solidity IDE (http://remix.ethereum.org), an open source tool for creating and testing smart contracts in the Ethereum blockchain infrastructure. As a cost metric we used the ‘gas’, a unit that measures the amount of computational effort required to execute certain operations. To calculate this cost in Euro, the amount of ‘gas’ is multiplied by the price of ‘gas’ in Ether (Ethereum's cryptocurrency). At the time of writing on 26 April 2018 the average price of ‘gas’ (https://etherscan.io/chart/gasprice) chosen was 14 Gwei (1 Gwei = 1 M Nanoether), and the exchange rate of Ether (https://etherscan.io/chart/etherprice) was 1 Ether = 545.298€.

### 4.1 Cost Analysis of the Basic Scheme

Table 2 presents the cost analysis of the contract for the basic scheme under different implementation approaches. The first row of this table shows the cost of the Contract 1 for the deployment process in the Ethereum. This contract stores a hash value (in a byte32 data type), accompanied by a timestamp, generated by the query results of the biomedical database and the requested query. The second and third rows show the cost of only one contract that is possible to provide the same functionality with Contract 1. These two contracts contain an array of elements, accompanied with a timestamp, and the only difference between them is the data types that are used to store the elements. Each element is a hash value that was generated from the query results of the biomedical database and the requested query. Thus, instead of creating a new contract to store the hash value for each new query request, we use only one contract and store the hash value in an array. In that way, the cost is lower because the cost for the creation of a smart contract is higher than the cost to add a variable (in byte32 or even string data type) on its storage. In all these experiments, we have used the Keccak hash algorithm with 256-bits length and in the case where we use string data type the hash values are encoded using Base64 as a more equitable solution. Finally, the only difference between the columns ‘Initial element’ and ‘Other elements’ is that the cost for only the first element that would be added in the array is higher because at this point the contract creates the required data structures.

**Table 2:** Cost analysis of the basic scheme.

Description	Deployment		Initial element		Other elements	
	Gas	Cost(€)	Gas	Cost(€)	Gas	Cost(€)
1. New contract* per query request	157,274	1.20	–	–	–	–
2. Only one contract* using array with byte32 data types	254,614	1.94	105,894	0.81	75,894	0.58
3. Only one contract* using array with string data types	441,335	3.37	148,362	1.13	118,362	0.90

* using 256-bits hash length

### 4.2 Cost Analysis of the Versioning Scheme

Table 3 presents the cost analysis of the versioning scheme under different implementation approaches. In the first row, we examine the case where we create a new contract with versioning of query results for each new query that is submitted to our service. This contract refers only to one query (more precisely, to the query's hash) and for that query it contains all the hash values of the query results, accompanied with timestamps, that correspond to that query since the day of its creation. The seconds row shows the cost of the contract that is presented in Contract 2 and stores all the hashes of the received queries and the corresponding hash results. The third and fourth rows show the cost of contracts that use string data types (in comparison with Contract 2 that uses byte32 data types) for different hash lengths, 256-bits and 512-bits accordingly. The usage of 512-bits hash functions currently requires to utilize only string data type because this amount of data can not fit into 32-bits variables. The cost of the ‘Initial element’ in case of the contract in the first row will only be needed when the first element that would be added in the array, but in the cases of 2^*nd*^ to 4^*th*^ rows this cost will be needed at any time where a new query is added in the contract.

**Table 3:** Cost analysis of the versioning scheme. All four cases support versioning.

Description	Deployment		Initial element		Other elements	
	Gas	Cost(€)	Gas	Cost(€)	Gas	Cost(€)
1. One contract per query	330,720	2.52	84,423	0.64	69,423	0.53
2. Single contract, 256-bits hash, and byte32 data types	361,160	2.76	86,797	0.66	71,797	0.55
3. Single contract, 256-bits hash, and string data types	914,243	6.98	131,332	1.00	116,332	0.89
4. Single contract, 512-bits hash, and string data types	914,243	6.98	157,605	1.20	142,605	1.09

### 4.3 Comparison of the Two Schemes

**Fig. 5:**
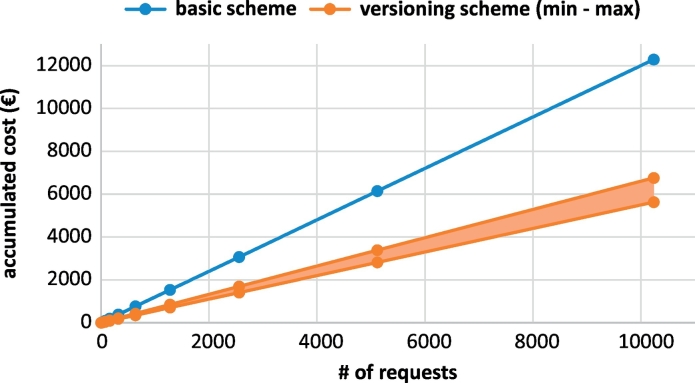
Accumulated cost of the basic (Contract 1) and versioning (Contract 2) scheme per number of requests.

The cost results in Table 2, Table 3 show that the versioning scheme spends less gas for higher functionalities than the basic scheme. This is due to the fact that the creation of a new contract costs to the notary service 157,274 Gas. However, when the service creates a single contract for all the queries (as in versioning scheme), the cost is 361,160 Gas to create the contract, an additional 86,797 Gas to add the hash of the first results set and then another 71,797 Gas to add hash for every subsequent results version. Fig. 5 shows the graph of accumulated cost for both schemes as a function of the number of requests placed to the database. In this graph, the cost of the basic scheme is given for the Contract 1 and the cost of the versioning scheme is given for the Contract 2. The slight variation in the cost of the versioning scheme is caused by the variations in the percentage of new queries submitted to the service. The cost is highest when all queries are distinct.

## 5 Implementation

Both schemes of the proposed service have been implemented to provide query notary services for two different biomedical databases, the CARRE risk factor reference repository [8] and the PubMed biomedical literature indexing database [9].

The CARRE risk factor reference repository is an open, online database collecting current high-level evidence on risk factors for the cardiorenal syndrome and related comorbidities. In this repository, risk factors are described in a structured way following the CARRE risk factor ontology [35]. Risk evidence descriptions are manually entered by authorized medical experts following a collaborative literature survey process by which appropriate medical publications of high level medical evidence are identified in PubMed and used to extract state-of-the-art medical evidence on risk factors related to cardiorenal disease. The resulting risk factor descriptions are available as Linked Data, following the Resource Description Framework (RDF) format (http://www.w3.org/TR/rdf-syntax), via an open access RDF repository. Currently the CARRE risk factor repository describes more than 100 different risk factors corresponding to 250 risk associations between more than 50 medical conditions related to cardiorenal disease as retrieved from 65 scientific publications. The CARRE reference database is intended to be queried by medical professional via a rich graphical interface but also by eHealth applications via an API.

PubMed is the online indexing service provided by the US National Library of Medicine, National Institutes of Health, comprising of more than 28 million citations for biomedical literature from life science journals and online books, PubMed is the largest collection of biomedical literature information and is considered one of the most significant sources of medical evidence for clinical research and also for clinical decision and evidence based medicine. In 2017, PubMed received 846 million queries by humans via the interactive web interface and 2.5 billion queries via API [36].

The proposed query notary service was implemented using the Ethereum blockchain infrastructure [7]. The use of the Ethereum blockchain infrastructure requires running an Ethereum node using the Geth client (version 1.8.7-stable). Smart contracts are implemented in the Solidity language (https://solidity.readthedocs.io), while a MongoDB database (https://www.mongodb.org) is deployed for the local storage of contracts and respective information.

The front-end of the prototype was implemented using JavaScript and Ajax asynchronous requests to establish communication with the CARRE RDF repository via its SPARQL end-point (https://devices.duth.carre-project.eu/sparql) and the PubMed via the *E*-utilities, a public API of the National Center for Biotechnology Information (NCBI) Entrez system and allow access to all Entrez databases (https://eutils.ncbi.nlm.nih.gov/entrez/eutils/). The Express web framework for Node.js (https://expressjs.com) was used to connect the front-end with the back-end of the query notary service.

**Fig. 6:**
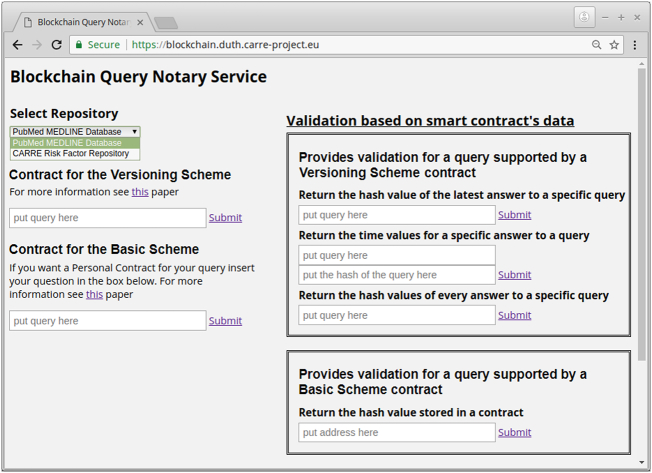
Snapshot of the blockchain query notary service.

**Fig. 7:**
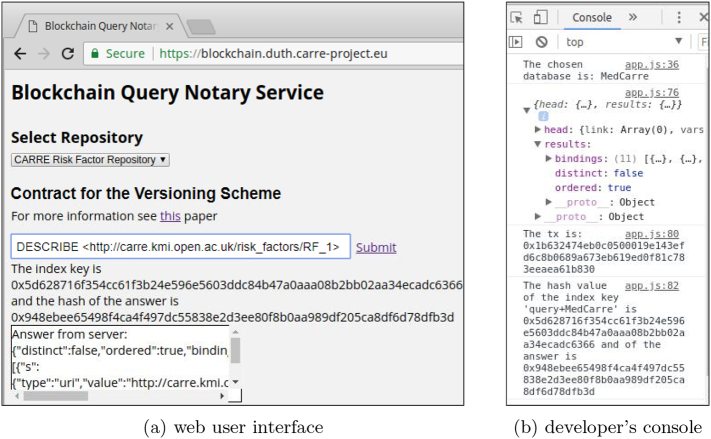
Contract deployment for the versioning scheme and the CARRE repository. (a) web user interface, (b) developer's console.

**Fig. 8:**
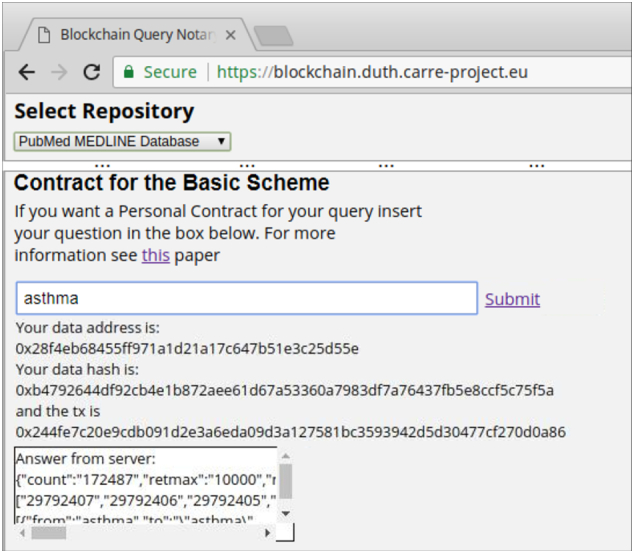
Contract deployment for the basic scheme and the PubMed MEDLINE database.

**Fig. 9:**
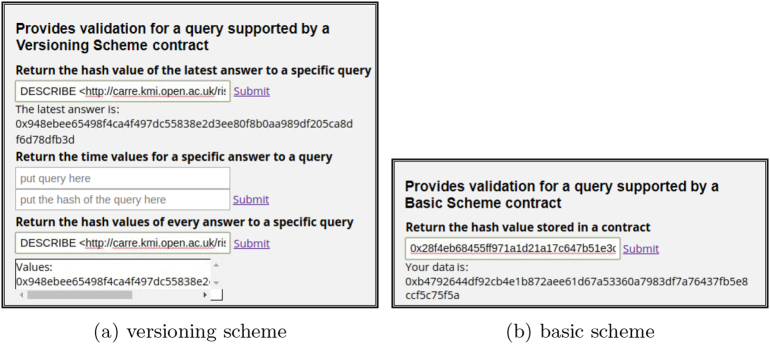
Verification process for the versioning and basic scheme through the front-end. (a) versioning scheme, (b) basic scheme.

Fig. 6 shows a snapshot of the blockchain query notary service as implemented for the CARRE risk factor repository and the PubMed MEDLINE database. When a new query is placed via the front end, the notary service communicates with the selected repository and for the basic scheme it creates a smart contract (Contract 1) out of the query and the returned results. Accordingly, for the versioning scheme it updates the appropriate values, if it is necessary, using the defined functions in the Contract 2. For the deployment of the smart contracts we have used Ethereum test networks and local networks. More precisely, the contracts for the basic and the versioning scheme have been deployed on the Ropsten test network (https://testnet.etherscan.io). The front end provides information on the status of the procedure and offers an area with input boxes that can be used for verification purposes. In the Fig. 7, Fig. 8, the contracts deployment processes for the versioning and basic scheme are shown. Furthermore, the verification process of both schemes is presented in Fig. 9. The demonstration of the proposed schemes along with supplementary material can be found at https://euclid.ee.duth.gr/demos/notary/.

## 6 Discussion

This paper proposes a query notary for biomedical data consumers (humans or programs alike) who need to retrieve accurate and certified data from reference biomedical databases. The proposed approach utilizes blockchain technology and is implemented using a real blockchain infrastructure for two different publicly available medical reference repositories.

The goal of this work is to provide a state of the art solution for ensuring integrity and non-repudiation of information retrieval operations when life-critical biomedical and clinical evidence is involved. The proposed biomedical database query notary service exhibits two different schemes to cover the different scenaria of use. The first scheme (*basic*) implements a query-response ledger by which the user attains a sealed proof that at a specific time a specific query has been placed in a biomedical database which returned specific results. This scheme can be used to seal the integrity and non-repudiation of a query and the retrieved results when a critical biomedical task depends on the specific query. An example could include the case where a biomedical literature database is queried to acquire state of the art medical evidence to carry out a systematic review or even to construct clinical guidelines. The second scheme (*versioning*) allows for non-reputable versioning of information retrieved from a dynamically evolving biomedical database at a number of occasions in time, always via the same query. This versioning scheme could be used to seal different versions of evolving medical evidence as retrieved from a biomedical database with content that is continually updated. An example could involve a health application which uses medical evidence as provided by a dynamic biomedical database for decision support in healthcare; using the versioning scheme the health application can ensure the integrity and non-repudiation of its knowledge base at any given point in time.

The results of this work show that a blockchain-based database query notarization service is viable and can support additional functionalities such as versioning of retrieved results over time. In the contexts of blockchains, there is a tradeoff between the complexity of the supported operations and the cost of the corresponding transactions. Our cost analysis experiments show that this cost is not so high and also it could be reduced using different implementation approaches of the same provided functionalities. Additionally, the proposed notary is realized following the approach of software-as-a-service, thus bringing the cost to the data consumer on a needs basis. A private blockchain network maintained by health regulators, such as healthcare establishments and medical research organizations (similar to that proposed in [33]) could be established to alleviate furthermore this cost.

The notary service is implemented as a lightweight wrapper and can support any type of biomedical data. The only requirement is that the service must be able to interact with the biomedical data source over some API. A limitation of current blockchain infrastructures is that the number of transactions per second is bounded. Consequently, the maximum rate of retrieval operations of the notary service is also bounded. Currently, blockchain infrastructures, including Ethereum, are advancing their technology to handle much larger transaction rates [37]. In the case biomedical database queries, one normally expects to require sealed proofs of queries and results, and thus invoke the proposed query notary service, only when medically and clinically critical retrieval operations are involved. Additionally, on the condition that blockchain infrastructures are widely available, the introduction of the notary service in a production environment is straightforward and there should be no significant barriers to its adoption. Finally, as the blockchain technology is increasingly applied, the corresponding legal issues will have to be addressed by the appropriate legislative bodies.

### 6.1 Future Directions

Potential future directions could be to support more functionalities (e.g. data consent usage) or to apply the proposed approach to different application domains, such as digital forensics and how the digital evidences could be ensured in a public verifiable way and free of leakages. Another interesting direction would be to consider smart contracts involving dynamic graph data (e.g. Linked Open Data cloud datasets), where the question is to combine certified sub-graphs (for example from different repositories) in order to validate larger, integrated data graphs.
